# Highly enantioselective synthesis of (*R*)-1,3-butanediol via deracemization of the corresponding racemate by a whole-cell stereoinverting cascade system

**DOI:** 10.1186/s12934-020-01384-3

**Published:** 2020-06-08

**Authors:** Han Zu, Jie Gu, Hui Zhang, Anwen Fan, Yao Nie, Yan Xu

**Affiliations:** 1 Key Laboratory of Industrial Biotechnology, Ministry of Education, School of Biotechnology, Jiangnan University, 1800 Lihu Road, Wuxi, 214122 China; 2 State Key Laboratory of Food Science and Technology, Jiangnan University, 1800 Lihu Road, Wuxi, 214122 China; 3 Suqian Industrial Technology Research Institute of Jiangnan University, Suqian, 223814 China

**Keywords:** Stereoselectivity, (*R*)-1,3-butanediol, Racemate, Whole-cell catalysis, Oxidation–reduction cascade

## Abstract

**Background:**

Deracemization, the transformation of the racemate into a single stereoisomeric product in 100% theoretical yield, is an appealing but challenging option for the asymmetric synthesis of optically pure chiral compounds as important pharmaceutical intermediates. To enhance the synthesis of (*R*)-1,3-butanediol from the corresponding low-cost racemate with minimal substrate waste, we designed a stereoinverting cascade deracemization route and constructed the cascade reaction for the total conversion of racemic 1,3-butanediol into its (*R*)-enantiomer. This cascade reaction consisted of the absolutely enantioselective oxidation of (*S*)-1,3-butanediol by *Candida parapsilosis* QC-76 and the subsequent asymmetric reduction of the intermediate 4-hydroxy-2-butanone to (*R*)-1,3-butanediol by *Pichia kudriavzevii* QC-1.

**Results:**

The key reaction conditions including choice of cosubstrate, pH, temperature, and rotation speed were optimized systematically and determined as follows: adding acetone as the cosubstrate at pH 8.0, a temperature of 30 °C, and rotation speed of 250 rpm for the first oxidation process; in the next reduction process, the optimal conditions were: adding glucose as the cosubstrate at pH 8.0, a temperature of 35 °C, and rotation speed of 200 rpm. By investigating the feasibility of the step-by-step method with one-pot experiment as a natural extension for performing the oxidation–reduction cascade, the step-by-step approach exhibited high efficiency for this cascade process from racemate to (*R*)-1,3-butanediol. Under optimal conditions, 20 g/L of the racemate transformed into 16.67 g/L of (*R*)-1,3-butanediol with 99.5% enantiomeric excess by the oxidation–reduction cascade system in a 200-mL bioreactor.

**Conclusions:**

The step-by-step cascade reaction efficiently produced (*R*)-1,3-butanediol from the racemate by biosynthesis and shows promising application prospects.

## Background

Chiral alcohols are widely used in the fields of medicine, food, chemicals, and pesticides due to their unique optical activity [1–3]. (*R*)-1,3-butanediol ((*R*)-1,3-BDO) is an important chiral alcohol used for the synthesis of pheromones, fragrances, and insecticides [4–6]. (*R*)-1,3-BDO is also a critical pharmaceutical intermediate, especially as a precursor for the synthesis of azetidinone derivatives, which in turn are key chiral intermediates for the synthesis of penem and carbapenem β-lactam antibiotics [7]. The demand for (*R*)-1,3-BDO has drastically increased as β-lactam antibiotics are the most used antibacterial agents in clinical practice worldwide; its production method has consequently been extensively studied [8, 9].

Currently, (*R*)-1,3-BDO is produced by chemical synthesis or microbial conversion. Regarding the chemical process, Boaz et al. [5] observed that ruthenium complexes of phosphine-aminophosphine produced (*R*)-1,3-BDO by the asymmetric reduction of its prochiral precursor 4-hydroxy-2-butanone (4H2B) with 81.8% enantiomeric excess (*ee*); Yasuo et al. [6] obtained 3-hydroxybutanal by acetaldehyde condensation, which was then hydrogenated to become (*R*)-1,3-BDO with the Raney Ni catalyst. However, the product obtained by the chemical route has a low optical purity, and preparation of the chemical catalyst is difficult and expensive. In addition, some dangerous reagents are usually necessary for chemical synthesis, and the reaction conditions are severe [10–12]. In contrast, the biocatalytic process presents the advantages of requiring mild reaction conditions, providing a high stereoselectivity, producing few by-products, and leaving no residual metals in the product [4, 11, 12]. Matsuyama et al. [13] isolated *Candida arborea* IAM 4147 and *Issatchenkia scutalata* IFO 10070 from soil samples and completed the conversion from 4H2B to (*R*)-1,3-BDO, with yields of only 37% and 48%; Zheng et al. [9] used *C. krusei* ZJB-09162 to transform 45 g/L of 4H2B into (*R*)-1,3-BDO through a fed-batch fermentation with 99% *ee,* and a yield of 83.9%. However, 4H2B is an expensive intermediate and is therefore unsuitable for large-scale industrial production. In order to solve this problem, Kataoka et al. [14] constructed a synthetic pathway by genetic engineering in *Escherichia coli* MG1655 lacIq to produce (*R*)-1,3-BDO from glucose: this process reached an optical purity of 98.5% *ee* and a 1,3-BDO concentration of only 9.05 g/L. Matsuyama et al. [15] also tried to complete the conversion from racemate to (*R*)-1,3-BDO using *C. parapsilosis* IFO 1396, achieving a 50% yield with 95% *ee*. The (*S*)-1,3-BDO in the racemate was dehydrogenated, while the (*R*)-1,3-BDO was preserved, which meant that the maximum yield of (*R*)-1,3-BDO was theoretically limited to 50%.

Although kinetic resolution is the most accessible method to obtain the enantiomerically pure alcohol from its racemate, through this process the maximum yield of the desired enantiomer is limited to 50% [16]. To overcome this drawback, a stereoinversion-based oxidation–reduction cascade is applied to the synthesis of chiral alcohols, reducing the waste of raw materials and energy, increasing the overall reaction yield, and reducing the time-consuming step of isolation of intermediates [17–19]. However, challenges involved in this process include screening absolutely stereospecific catalysts for selective oxidation and asymmetric reduction, respectively, connecting the oxidation and reduction steps synergistically without the need for isolating the intermediates, as well as controlling different reaction conditions between the oxidation and reduction steps [20, 21]. It is therefore critical to select two biocatalysts with opposite enantioselectivity for the oxidation–reduction stereoinversion and reduce the mutual influences in the cascade by optimizing reaction conditions [11, 22].

**Fig. 1:**
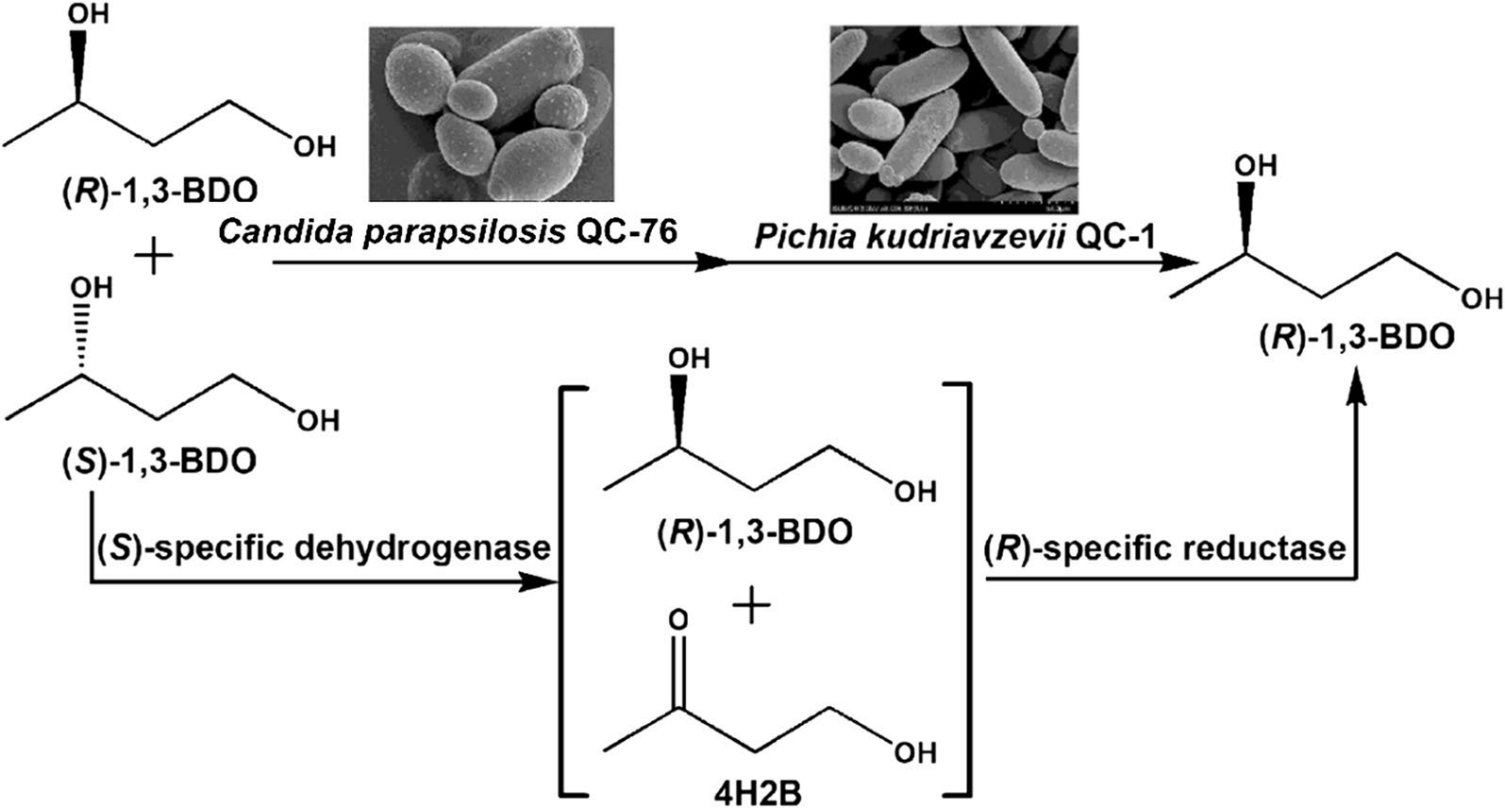
Schematic representation of the cascade oxidation–reduction system for (*R*)-1,3-BDO production from its racemate

In this work, we obtained *C. parapsilosis* QC-76 and *Pichia kudriavzevii* QC-1 with absolute stereoselectivity by screening more than 500 isolated strains from soil samples. Using two stereoselective strains, we aimed to develop an enantioselective cascade biocatalysis for the deracemization of racemic (*R, S*)-1,3-BDO to (*R*)-1,3-BDO (Fig. 1). *C. parapsilosis* QC-76 catalyzed the stereoselective oxidation and *P. kudriavzevii* QC-1 catalyzed the next asymmetric reduction, forming the whole cascade deracemization process. The two steps were conducted sequentially, producing optically pure (*R*)-1,3-BDO after the optimization of reaction conditions, including cosubstrate, pH, temperature, and rotation speed.

## Results

### Screening of microorganisms

**Fig. 2:**
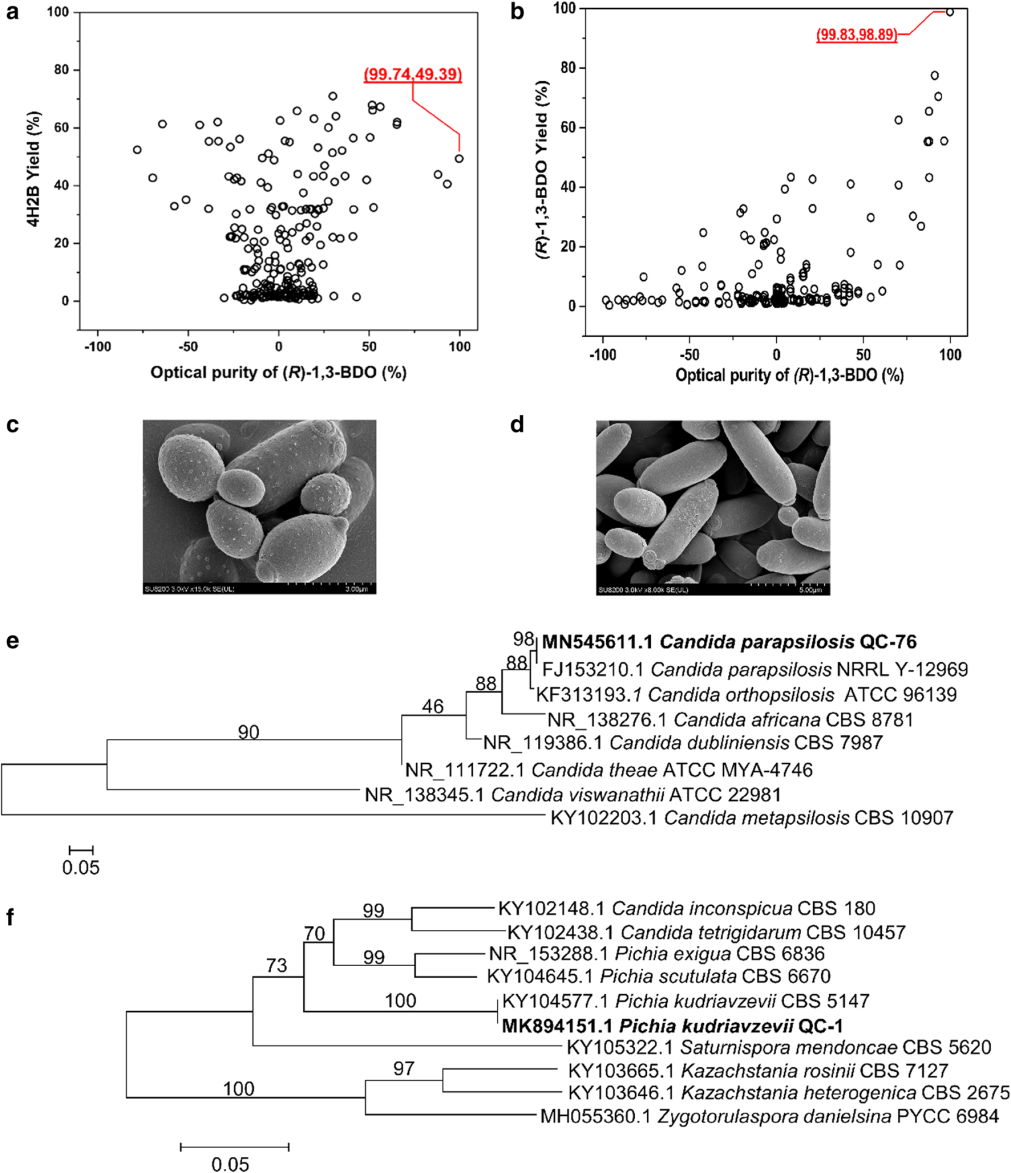
Functional screening, morphological observations and genes identification of target strains. **a** Screening of strains that transformed racemate into 4H2B; **b** screening of strains that transformed 4H2B to (*R*)-1,3-BDO; **c** field emission scanning electron microscope of strain QC-76; **d** field emission scanning electron microscope of strain QC-1; **e** phylogenetic tree of strain QC-76 based on 5.8S-ITS rDNA sequences; **f** phylogenetic tree of strain QC-1 based on 5.8S-ITS rDNA sequences

544 colonies were screened from soil samples for the two-step reaction. For the first oxidation step, racemate was chosen as the substrate, and we calculated the yield and the *ee* of (*R*)-1,3-BDO produced by the tested strains. As shown in Fig. 2a, most of the strains achieved a low *ee* (− 30 to 30%) and showed low specificity to racemate. In comparison, strain QC-76 was able to oxidize (*S*)-1,3-BDO into 4H2B and stereospecifically conserve the (*R*)-1,3-BDO in the racemate, providing 99.74% *ee* and a 49.39% yield. Strain QC-76 was therefore chosen for the oxidation step. Considering the reduction step, 4H2B was chosen as the substrate, and yields of (*R*)-1,3-BDO and its *ee* were compared. As shown in Fig. 2b, strain QC-1 provided yield and *ee* of (*R*)-1,3-BDO of 98.89% and 99.83%, respectively, and thus was chosen for the second step. These two strains, QC-76 and QC-1, would be used to construct a two-step reaction system for converting racemate into (*R*)-1,3-BDO.

### Strain identification

The morphologies of QC-76 and QC-1 were observed by field emission scanning electron microscope and both were preliminarily determined as yeast. Both strains presented elliptical and elongated cells, with buddings occurring at multiple sites (Fig. 2c, d).

To further identify the screened strains, we performed a 5.8S internal transcribed spacer (ITS) analysis. The 5.8S-ITS sequences of strains QC-76 and QC-1 were analyzed, and two phylogenetic trees were constructed. Sequences were deposited in GenBank under the accession numbers MN545611.1 and MK894151.1. As shown in Fig. 2e, f, strain QC-76 was closely clustered with *C. parapsilosis* NRRL Y-12969 (GenBank accession no. FJ153210.1), with a sequence identity of 98%; strain QC-1 was closely clustered with *P. kudriavzevii* CBS 5147 (GenBank accession no. CP028532.1), with a sequence identity of 100%. Based on the results of the phylogenetic analysis and phenotypic tests, the isolates were designated as *C. parapsilosis* QC-76 and *P. kudriavzevii* QC-1.

### Catalytic specificity of the identified strains

**Fig. 3:**
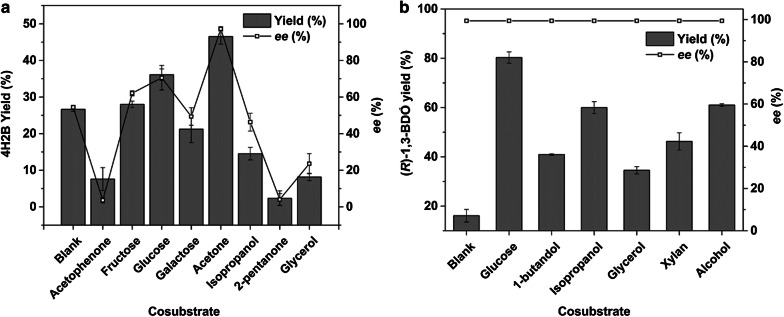
Effects of cosubstrates on single-step reactions. **a** Selective oxidation of racemate; **b** asymmetric reduction of 4H2B

In this work, to facilitate the whole cells-mediated oxidoreductions, different cosubstrates commonly used for cofactor regeneration were tested. For the oxidation step (Fig. 3a), when acetone was chosen as cosubstrate, the 4H2B yield reached 46.53% with 97.22% *ee*; regarding the reduction step (Fig. 3b), glucose as a cosubstrate resulted in a (*R*)-1,3-BDO yield of 80.3%, with 99.4% *ee*. Therefore, acetone and glucose were selected as the cosubstrates for the oxidation and reduction reactions, respectively.

**Fig. 4:**
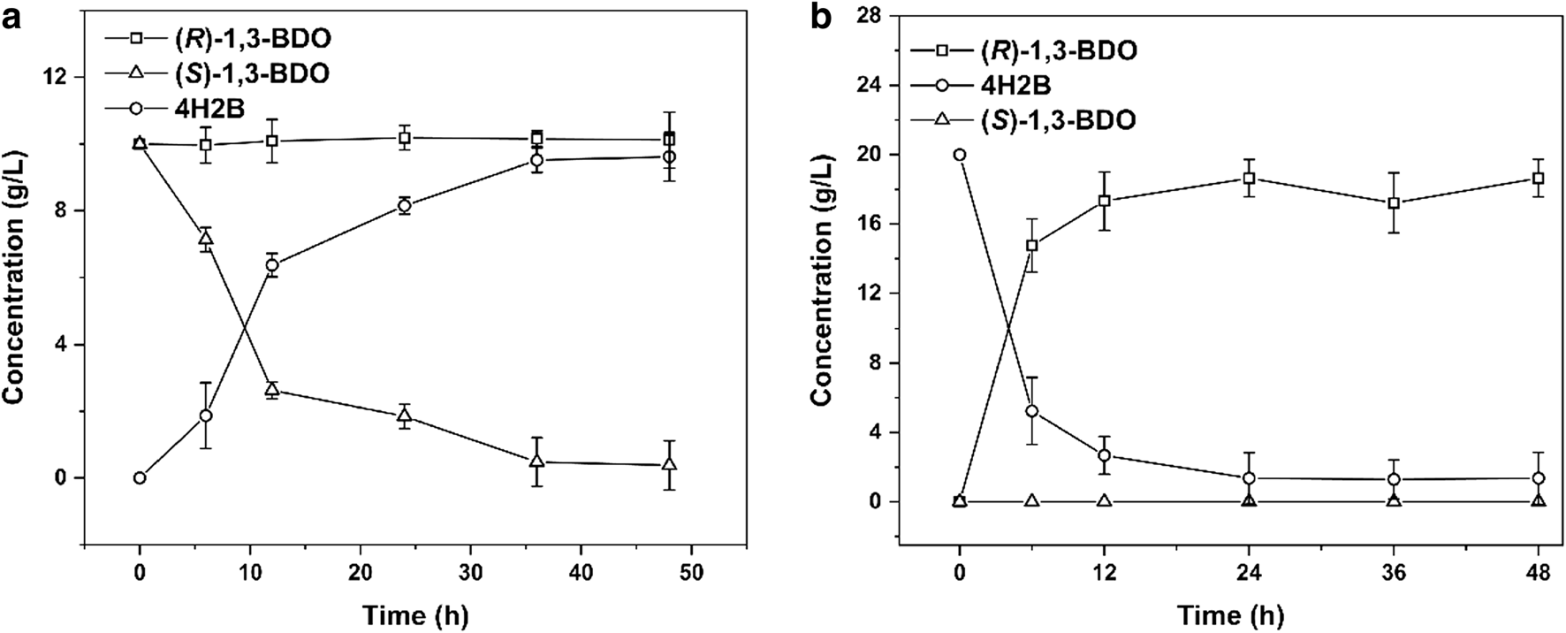
Time courses of single-step reactions. **a** Selective oxidation of racemate; **b** asymmetric reduction of 4H2B

In order to achieve a high optical purity in chiral alcohol production, the oxidation and reduction steps should present high stereoselectivity and yield. Two independent experiments were performed to verify the catalytic specificity of *C. parapsilosis* QC-76 towards (*S*)-1,3-BDO and of *P. kudriavzevii* QC-1 towards 4H2B. As shown in Fig. 4a, in the step catalyzed by *C. parapsilosis* QC-76, 10 g/L of (*S*)-1,3-BDO in the racemate were gradually reduced until almost zero, and the 4H2B concentration gradually increased from 0 g/L to 9.62 g/L. Meanwhile, the (*R*)-1,3-BDO concentration remained almost unchanged, indicating that (*S*)-1,3-BDO was dehydronated to 4H2B in a stereoselective manner. Figure 4b indicates that, in the step catalyzed by *P. kudriavzevii* QC-1, 20.00 g/L of 4H2B were transformed into 18.64 g/L of (*R*)-1,3-BDO with a 93.2% yield, and (*S*)-1,3-BDO was not detected. The asymmetric synthesis of (*R*)-1,3-BDO from 4H2B by *P. kudriavzevii* QC-1 was therefore also stereospecific, producing (*R*)-1,3-BDO with over 99% *ee*. Therefore, the two necessary steps involved in deracemization of racemic 1,3-BDO were achieved. To understand the compatibility between *C. parapsilosis* QC-76 catalyzing selective oxidation and *P. kudriavzevii* QC-1 catalyzing asymmetric reduction, effects of various reaction conditions on each single-step conversion were investigated, which provide the basis for further construction of stereoinverting cascade system.

### Effect of cosubstrate concentration on asymmetric oxidation/reduction

**Fig. 5:**
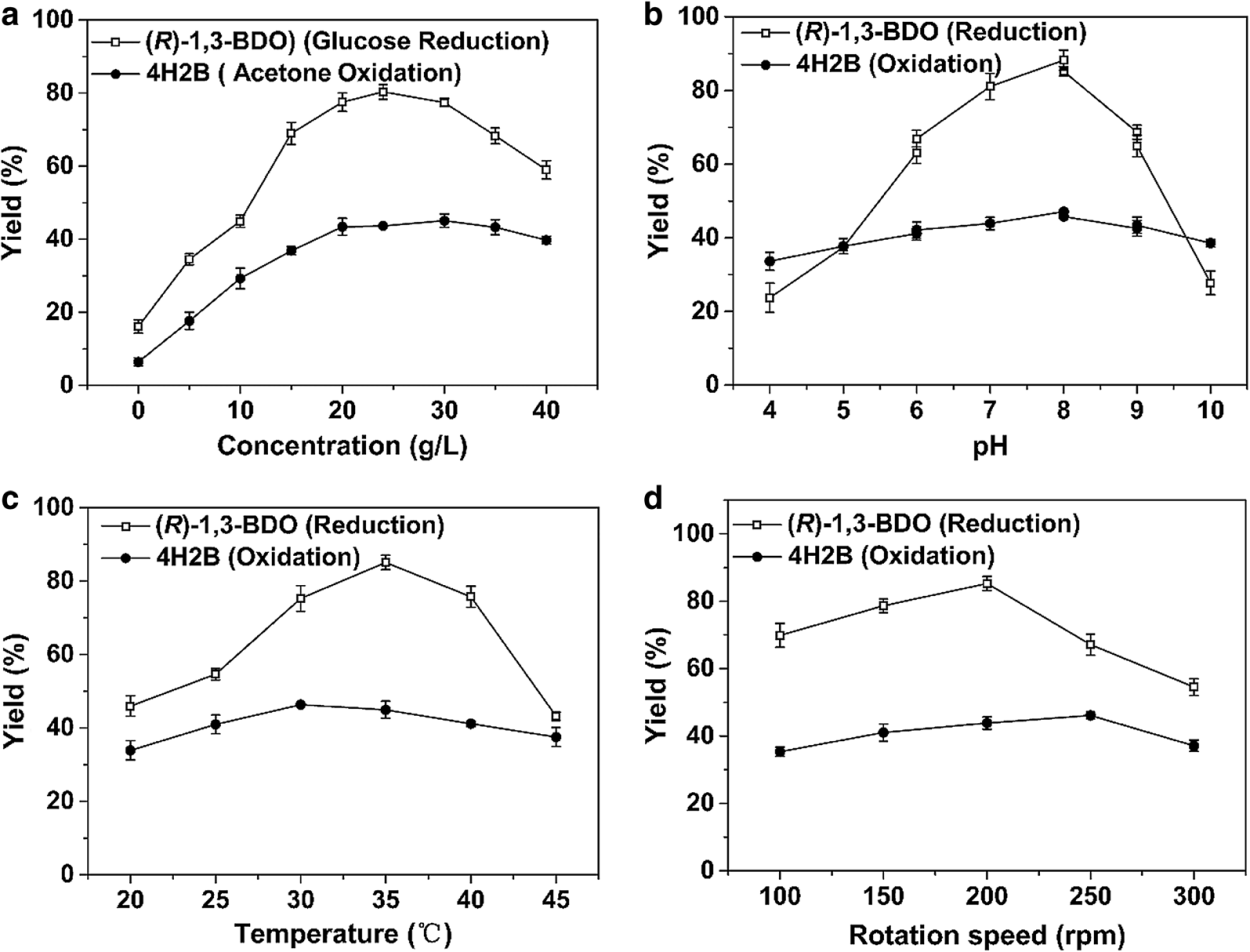
Effects of reaction conditions on oxidation from the racemate to 4H2B and on the reduction from 4H2B to (*R*)-1,3-BDO, respectively. **a** Effect of cosubstrate concentration on the asymmetric reaction, acetone used as the cosubstrate for oxidation from the racemate to 4H2B and glucose used as the cosubstrate for reduction from 4H2B to (*R*)-1,3-BDO; **b** effect of pH on the asymmetric oxidation and reduction, respectively; **c** effect of temperature on the asymmetric oxidation and reduction, respectively; **d** effect of rotation speed on the asymmetric oxidation and reduction, respectively

As shown in Fig. 5a, the use of 30 g/L acetone resulted in the best yield of 4H2B (45.06%) in the oxidation step. As the acetone concentration increased to 40 g/L, the yield decreased to 39.74%. Excess acetone has been reported to have a toxic effect on cells and to affect their catalytic activity [23]. Finally, the optimal mass ratio between acetone and racemate that yielded the most 4H2B was 3:1 (molar ratio = 4.7:1). For the reduction step, the (*R*)-1,3-BDO yield was the highest when glucose concentration was 24 g/L. When glucose concentration was increased above 24 g/L, the (*R*)-1,3-BDO yield rapidly decreased. The gluconic acid generated from glucose was probably accumulated, leading to a significant drop in pH that could affect cell survival and conversion efficiency [24]. Finally, the optimal mass ratio between glucose and 4H2B was 6:5 (molar ratio = 1:1.7), and the maximum (*R*)-1,3-BDO yield reached 80.3%.

### Effect of pH on asymmetric oxidation/reduction

As illustrated in Fig. 5b, the pH exerted a significant impact on the oxidation and reduction rates. Both oxidation and reduction steps were improved with a rise in pH from 4.0 to 8.0. Further increasing reaction pH, however, resulted in lower yields for both reactions. Finally, the optimum pH for the oxidation and reduction steps was determined to be 8.0, where the yields of the oxidation and reduction steps were 47.10% and 85.18%, respectively.

### Effect of temperature on asymmetric oxidation/reduction

As shown in Fig. 5c, temperature ranged from 20 to 45 °C, with 5 °C intervals. When temperature was below 25 °C, yields of the two-step reaction were generally low, indicating low enzyme activity at these temperatures. The optimal temperatures for the oxidation and reduction steps were 30 °C and 35 °C, respectively. When temperature increased to 45 °C, the yield of the oxidation reaction decreased to 37.52% and that for the reduction reaction decreased to 43.19%, which could indicate a partial inactivation of the cells’ enzymes.

### Effect of rotation speed on asymmetric oxidation/reduction

In our reaction, we found that when rotation speed was increased from 100 to 200 rpm, both yields were increased (Fig. 5d), indicating that mass transfer had a strong influence on the reaction process. Considering the oxidation step, the product yield showed little decrease when the rotation speed was above 250 rpm. However, in the reduction step, the product yield showed a big drop when rotation was increased to 300 rpm, reaching only 54.51%. Finally, the optimal rotation speed for the oxidation reaction was 250 rpm, while for the reduction reaction it was 200 rpm.

### Preparative scale cascade bioconversion of racemate to (*R*)-1,3-BDO

**Fig. 6:**
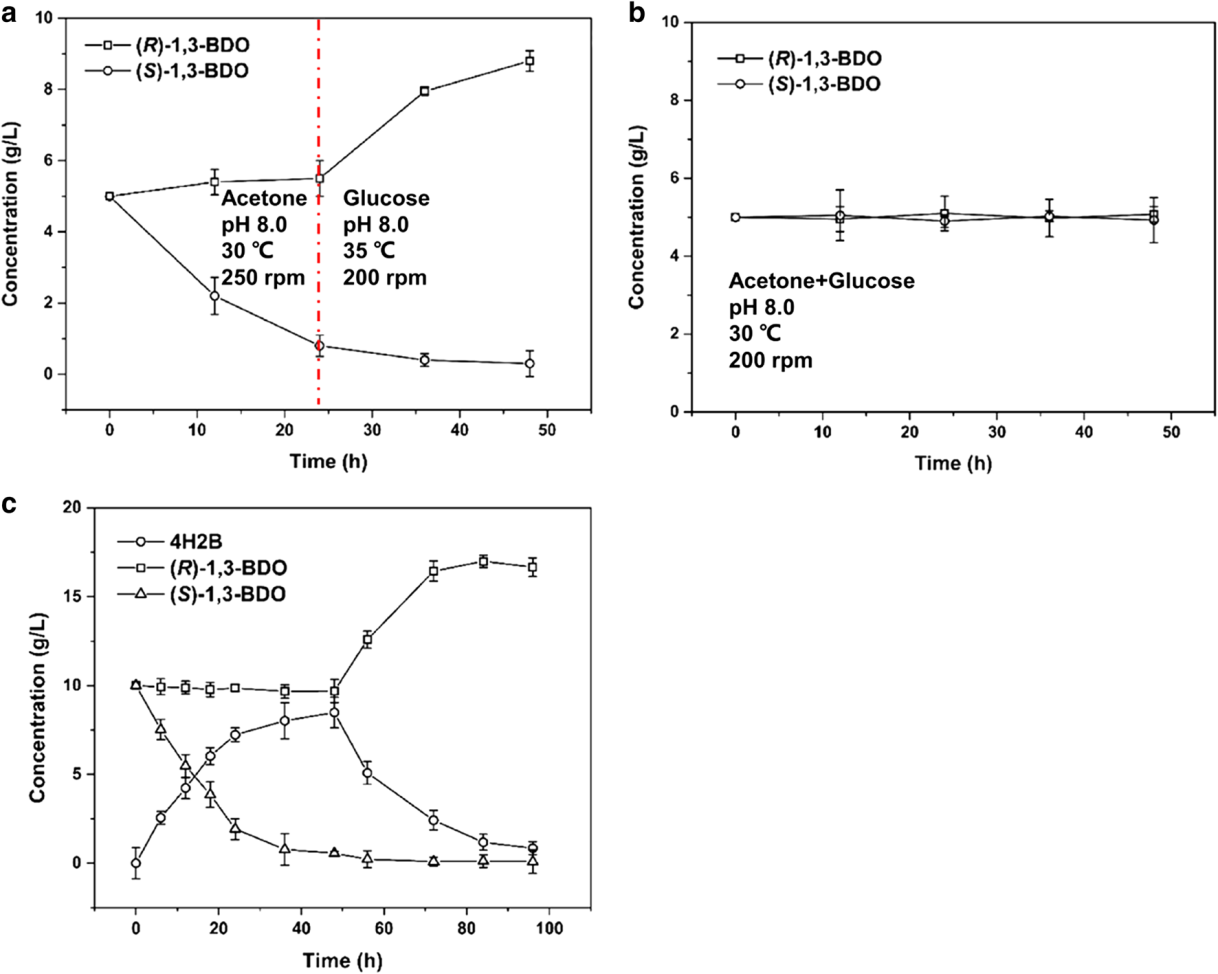
Comparison of catalytic processes and scale-up of reaction system. **a** Step-by-step catalysis for the deracemization; **b** one-pot catalysis for the deracemization; **c** conversion of racemate to (*R*)-1,3-BDO in a 500-mL bioreactor

For constructing the stereoinverting cascade system, the conversion efficiency of the step-by-step approach was investigated, with one-pot method as a natural extension of the step-by-step experiment for performing the oxidation–reduction cascade. As shown in Fig. 6a, after a 48-h reaction, through the step-by-step approach we obtained 8.8 g/L of (*R*)-1,3-BDO with 88% yield, and the *ee* of (*R*)-1,3-BDO was improved to 96.7%. However, when using the second approach (one-pot reaction) (Fig. 6b), the concentrations of (*R*)-1,3-BDO and (*S*)-1,3-BDO remained almost constant, the *ee* of (*R*)-1,3-BDO was close to zero, and this process therefore failed to complete deracemization. These results showed that there is a difference between the optimal oxidation and reduction conditions. In addition, the two strains also affect each other throughout the catalytic reaction in the one-pot system. Therefore, the step-by-step approach was adopted for this cascade process. *C. parapsilosis* QC-76 was firstly added to catalyze the oxidation reaction with acetone as the cosubstrate, then the cells were removed by centrifugation after the oxidation reaction ((*S*)-1,3-BDO in the racemic substrate was converted to the intermediate 4H2B); subsequently *P. kudriavzevii* QC-1 was added to catalyze the reduction reaction with glucose as the cosubstrate (the intermediate 4H2B was converted to (*R*)-1,3-BDO).

According to the optimal condition results (cosubstrate, pH, temperature, and rotation speed) previously obtained, the reaction system was scaled up to a 200-mL working volume in a 500-mL reactor. Since the optimum pH was the same for the oxidation and the reduction steps (pH 8.0), the pH was maintained at 8.0 by the addition of 1 M sodium hydroxide during the reaction. As shown in Fig. 6c, 10 g/L of (*S*)-1,3-BDO (20 g/L racemate substrate) were converted into 8.75 g/L of 4H2B by *C. parapsilosis* QC-76 after a 48-h reaction; subsequently, *C. parapsilosis* QC-76 cells were removed and *P. kudriavzevii* QC-1 cells were added to the reaction, and 4H2B was transformed into (*R*)-1,3-BDO with the final process yield 83.35% and a 99.5% *ee*.

## Discussion

Stereoinversion-based deracemization is an important reaction route for asymmetric synthesis of various optically pure compounds, especially chiral pharmaceutical intermediates. Combination of suitable biocatalysts with desired stereospecificity and compatible catalytic properties provides a favorable alternative for constructing cascade biosystem catalyzing stereoinversion-based deracemization. However, screening biocatalysts with extremely high stereoselectivity and constructing a facile and efficient deracemization pathway are often the main challenges in the development of the required biological processes [9]. In this study, 544 colonies were isolated from the soil samples mostly collected from the regions close to pharmaceutical and chemical plants, which provide a high possibility of obtaining desired functional microorganisms performing activity towards relevant chemical compounds. By simultaneously evaluating the activity and stereoselectivity of candidate strains towards 4H2B or racemic 1,3-BDO, the strains QC-76 and QC-1 with the high conversion rate and stereospecificity (> 99% *ee*) were screened for the oxidation and reduction reaction, respectively. It is worth to note that the obtained strains exhibited higher stereospecificity towards their corresponding reactions, than most of the previously reported stereospecific whole-cell biocatalysts, such as *C. parapsilosis* IFO 1396 (95% ee), *Geotrichum candidum* (95% *ee*), *C. intermedia* IFO 0761 (76% *ee*), *Kluyveromyces lactis* IFO 1267 (93% *ee*), *C. utilis* lAM 4277 (95% *ee*), and *Hansenula polymorpha* ATCC 26012 (85% *ee*) [15, 25]. In addition, these two strains were compatible for constructing a whole-cell stereoinverting cascade system. Through the cascade biocatalysis, the optical purity of the product (*R*)-1,3-BDO could be achieved over 99%. Based on the taxonomical identification involving microbial morphology and sequence of 5.8S-ITS rDNA region, the strain QC-76 was identified as *C. parapsilosis* QC-76, and the strain QC-1 was identified as *P. kudriavzevii* QC-1. Of the two functional microorganisms, *P. kudriavzevii* QC-1 was a novel strain discovered from natural source for catalyzing stereoselective reaction.

In order to further increase the bioconversion efficiency, we investigated the effects of reaction components on the yield and optical purity of the objective product. For stereoselective redox reaction, deficiency of necessary cofactor and its regeneration generally leads to a low substrate concentration or a low conversion efficiency [26]. To facilitate cofactor regeneration, various cosubstrates have been employed for recycling cofactors, with largely varied effects in terms of product yield and *ee* [27, 28]. For the first step of oxidation, compared with other cosubstrates, such as glucose (75.05% *ee*), fructose (61.84% *ee*), and isopropanol (41.28% *ee*), acetone was more favorable for giving the optical purity of 97.79% *ee*. In the reaction, the added acetone promoted the complete and stereospecific conversion from (*S*)-1,3-BDO in the racemic substrate to 4H2B by *C. parapsilosis* QC-67 with the yield close to 50%. Therefore, (*S*)-1,3-BDO in the racemic substrate was specifically oxidized to the intermediate 4H2B while (*R*)-1,3-BDO was retained during the deracemization process, which is essential to improve the optical purity of the final (*R*)-1,3-BDO (> 99% *ee*) and enables the construction of a deracemization reaction route to meet the requirements of producing chiral building block with high optical purity. For the second step of reduction, glucose was selected as the suitable cosubstrate, providing reducing hydrogen to the conversion of oxidized cofactor into the corresponding reduced form and favoring the asymmetric reduction from the intermediate 4H2B to the final product (*R*)-1,3-BDO.

In the process of microbial cells-mediated stereoselective transformation, temperature and pH are also important influencing factors for reactions. Change of temperature directly affects cell stability and enzyme activity, leading to changes of reaction rate and equilibrium [29]; switch of pH value of reaction system influences not only cell activity and dissociation state of functional groups in enzyme active site, but also the cofactor-involved electron transfer system [29, 30]. By optimization of these reaction conditions, the optimum temperatures for the first oxidation and the second reduction were determined as 30 °C and 35 °C, respectively; the optimum pH value for both oxidation and reduction steps was determined as pH 8.0, indicating the catalytic compatibility of the two whole-cell biocatalysts. In addition, rotation speed of a reaction vessel affects diffusion and mass transfer of the substrate and product during catalytic reactions [31]. The optimal rotation speed for the oxidation reaction was 250 rpm, while for the reduction reaction it was 200 rpm. It was presumed that the excessively fast rotation speed increased the availability of dissolved oxygen in the reaction solution, resulting in a negative influence on the reduction reaction [32]. By investigating the effects of various reaction conditions on each single-step conversion, we not only obtained the optimized conditions for conducting the oxidation–reduction cascade, but also understood the compatibility between *C. parapsilosis* QC-76-catalyzed oxidation and *P. kudriavzevii* QC-1-catalyzed reduction for further construction of suitable cascade system.

In this work, for constructing the stereoinverting cascade system, the feasibility of the step-by-step approach was investigated, with one-pot method as a natural extension of the step-by-step experiment for performing the oxidation–reduction cascade. Consequently, the step-by-step approach was adopted for this cascade process to avoid the mutual influence between the two microorganisms on the stereoinverting conversion. To facilitate the stereoinverting cascade reaction by the two whole-cell biocatalysts, the cells of *C. parapsilosis* QC-67 were removed after the first oxidation step to prevent the negative effects of this strain on the second reduction step of 4H2B to (*R*)-1,3-BDO initiated by adding *P. kudriavzevii* QC-1 cells, while isolation of the intermediate 4H2B and change of reaction buffer were avoided, which minimized the loss of necessary reaction components, especially the substrate, the intermediate, and the product, and made the operation process more feasible and easier. Taken together, two whole-cell biocatalysts, *C. parasilosis* QC-76 and *P. kudriavzevii* QC-1, were characterized and used in the deracemization cascade synthesis of (*R*)-1,3-BDO. After optimizing reaction conditions, 20 g/L racemate was converted into 16.67 g/L (*R*)-1,3-BDO in a 200-mL reaction with absolute enantioselectivity (> 99% *ee*).

Concerning oxidation–reduction cascade, synthesis of other chiral alcohols, such as (*R*)-1-phenyl-1,2-ethanediol, has been reported to be achieved through stereoinversion-based deracemization by enzymatic catalysis [21]. For the coupled enzymes sequentially catalyzing cascade reaction, it would be necessary to keep the functional enzymes working in a compatible and synergistic way [21]. In this work, novel whole-cell biocatalysts were obtained to be capable of catalyzing stereoinverting cascade deracemization, producing (*R*)-1,3-BDO of high yield and optical purity. Based on the reaction route of cascade oxidation–reduction stereoinversion (Fig. 1), associating with the time courses of involved reactions (Fig. 4), we presumed that *C. parapsilosis* QC-76 would contain an (*S*)-specific dehydrogenase catalyzing selective oxidation from (*S*)-1,3-BDO to 4H2B and *P. kudriavzevii* QC-1 would contain an (*R*)-specific carbonyl reductase catalyzing asymmetric reduction from 4H2B to (*R*)-1,3-BDO. The stereoselective oxidoreductases from the newly isolated strains will be further identified for constructing efficient enzymatic cascade system.

## Conclusions

To the best of our knowledge, this is the first time that optically pure (*R*)-1,3-BDO was produced from the corresponding low-cost racemate by a cascade pathway with minimal substrate waste. Additionally, the proposed process is easy to operate since it relies on whole-cell biocatalysis and does not require isolation of intermediate. The deracemization cascade combining *C. parapsilosis* QC-76-mediated oxidation and *P. kudriavzevii* QC-1-mediated reduction provided high yield and stereochemical selectivity for (*R*)-1,3-BDO synthesis. Therefore, this work represents a discovery of novel whole-cell biocatalysts capable of performing stereoinversion in a facile and efficient manner, which shows much promise for further industrial applications.

## Methods

### Chemicals

Racemic 1,3-BDO, 4H2B, (*R*)-1,3-BDO and (*S*)-1,3-BDO were purchased from J&K Scientific Co. (Beijing, China). All other medium components and chemical reagents were purchased from the Sinopharm Chemical Reagent Company (Shanghai, China).

### Analytical methods

The optical purity of (*R*)-1,3-BDO was determined by HPLC using a Chiralcel OB-H column (250 mm × 4.6 mm; Daicel Chemical Industries, Tokyo, Japan), with a mobile phase of hexane–isopropanol (19:1) at a flow rate of 1.0 mL/min. The column temperature was 40 °C, and we used a spectronic detector (ultraviolet, λ = 220 nm. Shimazu, Kyoto, Japan) [25].

The (*R*)-1,3-BDO yield was determined by GC-flame ionization using an Econo Cap-Wax column (30 m × 250 μm × 0.25 μm; Alltech, Chicago, USA) and N_2_ as the carrier gas at a flow rate of 40 mL/min. The inlet and detector temperatures were 230 °C and 220 °C respectively, the column temperature was 225 °C, and the injection volume was 0.5 μL [33].

### Screening and cultivation

The medium used for cultivating isolated strains contained the following components: 2.0 g/L (NH_4_)_2_SO_4_, 1.0 g/L KH_2_PO_4_, 5.0 g/L yeast extract, 10.0 g/L tryptone, and 10.0 g/L glucose (pH 7.0; solid medium also received 20.0 g/L agar) [25].

More than 100 soil samples were mostly collected from the regions close to pharmaceutical factories and chemical plants, etc. Soil as a source of microbial strains was diluted with sterile water at 1:100 (w/v), coated on screening plate medium and incubated at 30 °C for 24 h. Single colonies were then picked, incubated in a 48-well plate with cultivating medium, and cultured at 30 °C for 48 h [34]. These cultures were centrifuged at 7000×*g* for 10 min, and 10 g/L 4H2B or racemic 1,3-BDO were then added to each well. The reaction was then carried out at 30 °C and 200 rpm for 48 h. The resulting cultures were centrifuged at 18,514×*g* for 5 min, the supernatant was obtained, saturated with sodium chloride and then extracted with 2 volumes of ethyl acetate for 10 min. The extracts were divided into two parts: one was dried using anhydrous Na_2_SO_4_ for gas chromatography (GC) analyses [33], and the other had the ethyl acetate removed by nitrogen blowing and acetylation with acetyl chloride in an ice bath for 10 min, followed by resuspension of the reaction mixture in 2-propanol for optical purity analysis by high-performance liquid chromatography (HPLC) [25].

Yeast extract-peptone-dextrose (YPD) medium was used for cultivating strains QC-76 and QC-1, containing 5.0 g/L yeast extract, 10.0 g/L tryptone, and 10.0 g/L glucose (pH 7.0; solid medium received 20.0 g/L agar). The strains were cultivated at 30 °C with 200 rpm shaking for 48 h. Cells were collected, washed with a 0.9% sodium chloride solution, and used directly for biotransformation.

### Identification of microorganisms

The morphologies of strains QC-76 and QC-1 were observed by scanning electron microscopy (SU8220; Hitachi, Tokyo, Japan) after growth on YPD agar for 2 days.

The isolated strains were taxonomically identified by sequencing the 5.8S-ITS rDNA region. We amplified these sequences with primers pITS1 (5′-TCCGTAGGTGAACCTGCCG-3′) and pITS4 (5′-TCCTCCGCTTATTGATATGC-3′) [35]. The obtained 5.8S-ITS regions were determined and aligned with the reference sequences retrieved from the GenBank database Clustal W [36]. Related sequences were obtained from the GenBank database using the Basic Local Alignment Search Tool (BLAST). The Molecular Evolutionary Genetics Analysis (MEGA) 6 software v. 6.0 was applied for the calculation of evolutionary distances, and a phylogenetic tree was constructed using the neighbor-joining method [37].

### Verification of each single-step reaction by functional strain

The verification experiments included the *C. parapsilosis* QC-76-catalyzed oxidation reaction and the *P. kudriavzevii* QC-1-catalyzed reduction reaction. According to previous reports and pre-tests [38, 39], we evaluated the effect of several cosubstrates, such as acetophenone, fructose, glucose, galactose, acetone, isopropanol, 2-pentanone, and glycerol in the oxidation step; for the reduction step, we evaluated glucose, 1-butanol, glycerol, xylan, and ethanol. The conditions for the oxidation reaction included 20 g/L of racemate, 1.5 g of wet strain QC-76, 10 g/L of acetone, 30 °C, 200 rpm, pH 7.0; regarding the reduction reaction, conditions included 20 g/L of 4H2B, 1.5 g of wet strain QC-1, 20 g/L of glucose, 30 °C, 200 rpm, and pH 7.0. All experiments were carried out in 10-mL reaction systems, and the contents of 4H2B, (*R*)-1,3-BDO, and (*S*)-1,3-BDO were detected throughout the reactions.

### Optimization of single-step reaction conditions

Cosubstrates, pH, temperature, and rotation speed were optimized separately for the oxidation and reduction reactions. The temperature range was 20–45 °C, with 5 °C intervals; the pH range was 4.0–10.0, with pH 1.0 intervals; the rotation speed range was 100–300 rpm, with 50-rpm intervals.

### Stereoinverting cascade for deracemization of 1,3-BDO to (*R*)-1,3-BDO

Two approaches for performing the oxidation–reduction cascade in the 10-mL reaction system were conducted. In the first approach (step-by-step manner), 2 g of wet strain QC-76 was used to conduct the first oxidation reaction with acetone as the cosubstrate under pH 8.0 at 30 °C and 250 rpm for 24 h, then the strain was discarded, and finally the second step was conducted by adding 2 g of wet strain QC-1 with glucose as the cosubstrate under pH 8.0 at 35 °C and 200 rpm for 24 h. In the second approach (one-pot reaction), 2 g of wet strains QC-76 and QC-1 were simultaneously added into the one-pot reaction system comprising racemic substrate, acetone, and glucose under pH 8.0 at 30 °C and 200 rpm in order to perform the oxidation–reduction cascade reaction at the same time. According to the obtained results, the cascade reactions were scaled up to a 500-mL bioreactor (EnzyR 500 mL; T&J, Shanghai, China) with 200 mL working volume. First, 20 g/L of the racemate and 35 g of wet strain QC-76 were added to the reaction, and pH was maintained at 8.0 with 1 M sodium hydroxide. The concentrations of 4H2B, (*R*)-1,3-BDO, and (*S*)-1,3-BDO were detected during the conversion process. When the (*S*)-1,3-BDO was mostly transformed into 4H2B, the QC-76 cells were discarded by centrifugation, and 35 g of wet strain QC-1 and the glucose were then added for the transformation from 4H2B to (*R*)-1,3-BDO.

## Data Availability

We declared that materials described in the manuscript, including all relevant raw data, will be freely available to any scientist wishing to use them for non-commercial purposes, without breaching participant confidentiality.

## Abbreviations

*ee*: Enantiomeric excess
1,3-BDO: 1,3-Butanediol
4H2B: 4-Hydroxy-2-butanone
ITS: Internal transcribed spacer
GC: Gas chromatography
HPLC: High-performance liquid chromatography
YPD: Yeast extract-peptone-dextrose
BLAST: Basic Local Alignment Search Tool
MEGA: Molecular Evolutionary Genetics Analysis
